# Supplementary material for: Interfacial interaction and intense interfacial ultraviolet light emission at an incoherent interface
Source: Nat Commun. 2023 May 15;14:2788. doi: 10.1038/s41467-023-38548-9 (PMC10185513; doi:10.1038/s41467-023-38548-9)
Supplement: Supplementary file 1 — Supplementary Information [file 41467_2023_38548_MOESM1_ESM.pdf]

Supplementary Information for

Interfacial interaction and intense interfacial ultraviolet light emission at an incoherent interface

Xuexi Yan[1], Yixiao Jiang[1], Qianqian Jin[2], Tingting Yao[1], Weizhen Wang[1], Ang Tao[1], Chunyang Gao[1], Xiang Li[1], Chunlin Chen[1,3], Hengqiang Ye[3] & Xiu-Liang Ma[1,4,5]

[1]Shenyang National Laboratory for Materials Science, Institute of Metal Research, Chinese Academy of Sciences, School of Material Science and Engineering, University of Science and Technology of China, Shenyang 110016, China

[2] Center for the Structure of Advanced Matter, School of Electronic Engineering, Guangxi University of Science and Technology, Liuzhou 545006, China

[3]Ji Hua Laboratory, Foshan 528200, China

[4] Bay Area Center for Electron Microscopy, Songshan Lake Materials Laboratory, Dongguan 523808, China

[5] Institute of Physics, Chinese Academy of Sciences, Beijing 100190, China

Corresponding author: Chunlin Chen (clchen@imr.ac.cn), Xiu-Liang Ma (xlma@imr.ac.cn)

Xuexi Yan and Yixiao Jiang contributed equally to this work.

Supplementary Figure S1

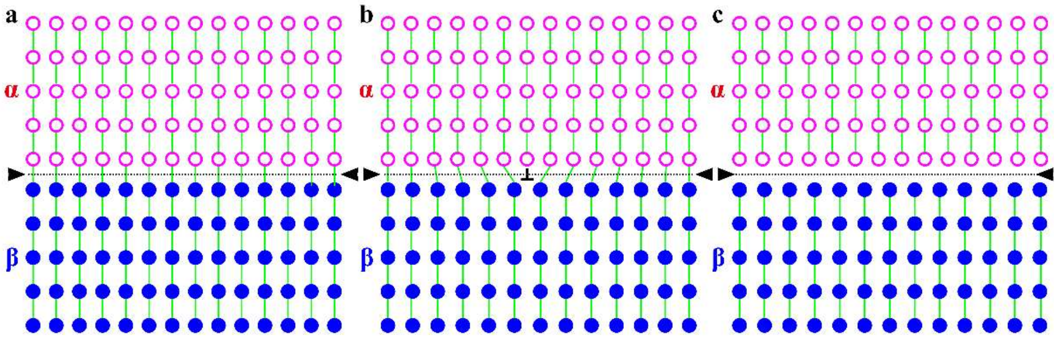

Supplementary Figure S1 | Schematic diagrams showing the atomic structure of interfaces. (a) Coherent interface. The coherent interface with a small mismatch has a perfect atom-by-atom matching interfacial structure since the interfacial mismatch is accommodated by the elastic deformation of two adjacent crystal lattices. **(b)** Semi-coherent interface. The semi-coherent interface with a moderate mismatch compensates the interfacial mismatch by introducing periodic misfit dislocations. **(c)** Incoherent interface. The very large lattice mismatch cannot be compensated by misfit dislocations. The adjacent crystals are rigidly stacked together by maintaining their respective lattices without deformation. α and β represent two adjacent crystals, respectively.

Supplementary Figure S2

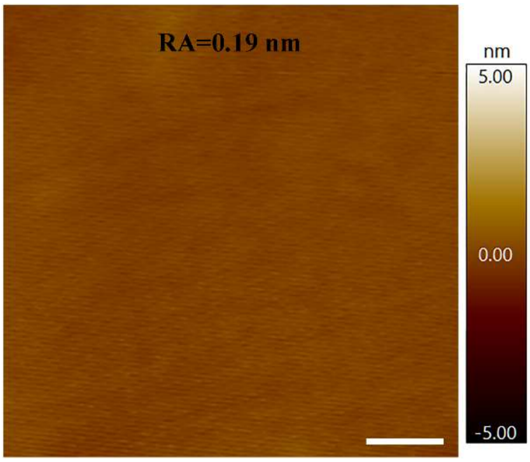

Supplementary Figure S2 | Topographic AFM image obtained by the tapping mode showing the surface morphology of the AlN thin film. The surface roughness of the film is about 0.19 nm. RA represents roughness average. Scale bar, 200 nm.

Supplementary Figure S3

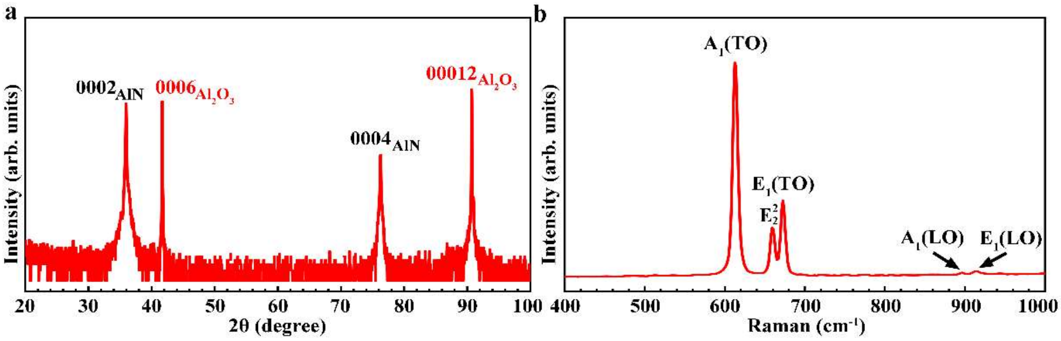

Supplementary Figure S3 | (a) Out-of–plane HRXRD pattern of the as-prepared AlN thin film on the Al_2O_3 (0001) substrate. **(b)** Raman spectrum of the as-prepared AlN thin film. As-prepared AlN thin film is composed of wurtzite AlN and has a good crystallinity. A_1(TO), E_2^2, and E_1(TO), A_1(LO), and E_1(LO) represent different first-order Raman active modes of AlN.

Supplementary Figure S4

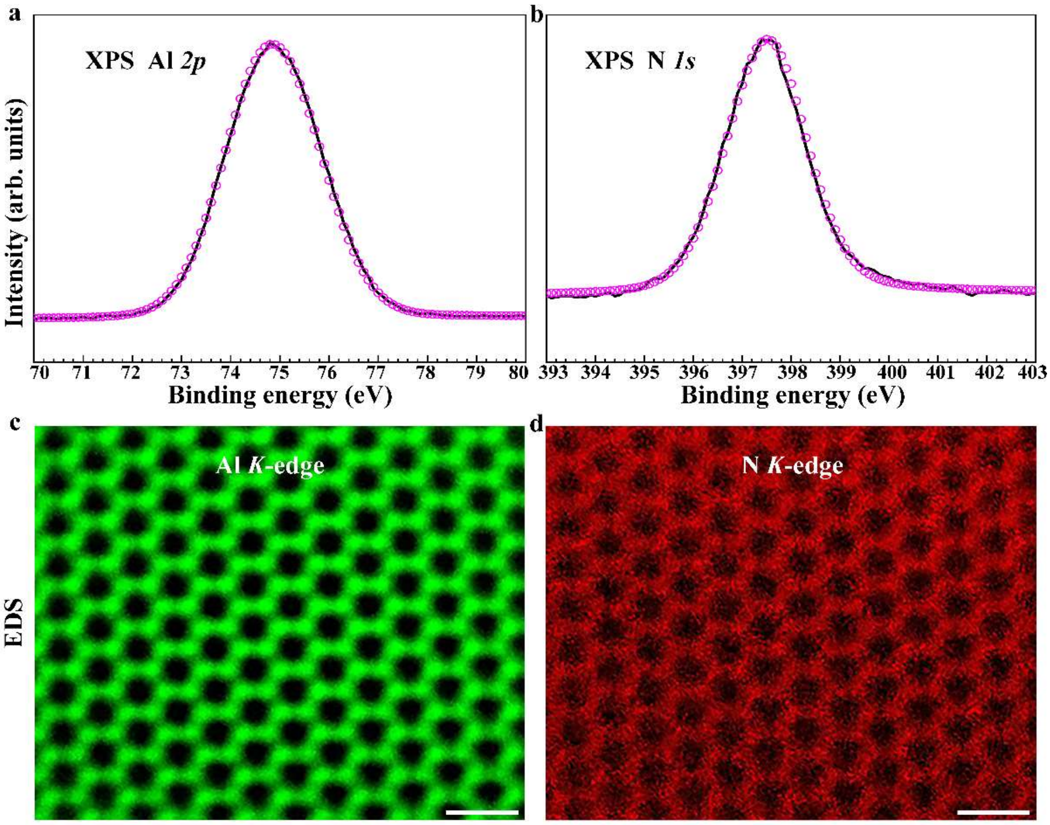

Supplementary Figure S4 | (a,b) XPS spectra of Al *2p* and N *1s* on the AlN film. Both the Al *2p* and N *1s* peaks are very smooth, indicating that the AlN thin film satisfies the stoichiometric ratio. The O *1s* XPS spectrum cannot be detected on the AlN film. **(c,d)** Atomic-resolution EDS mappings of the AlN film obtained along the [0001] zone axis. Al and N atoms are homogeneously distributed in the film. Quantitative analyses using the XPS and EDS techniques reveal that the AlN film has a good stoichiometric ratio. Scale bar, 5 Å.

Supplementary Figure S5

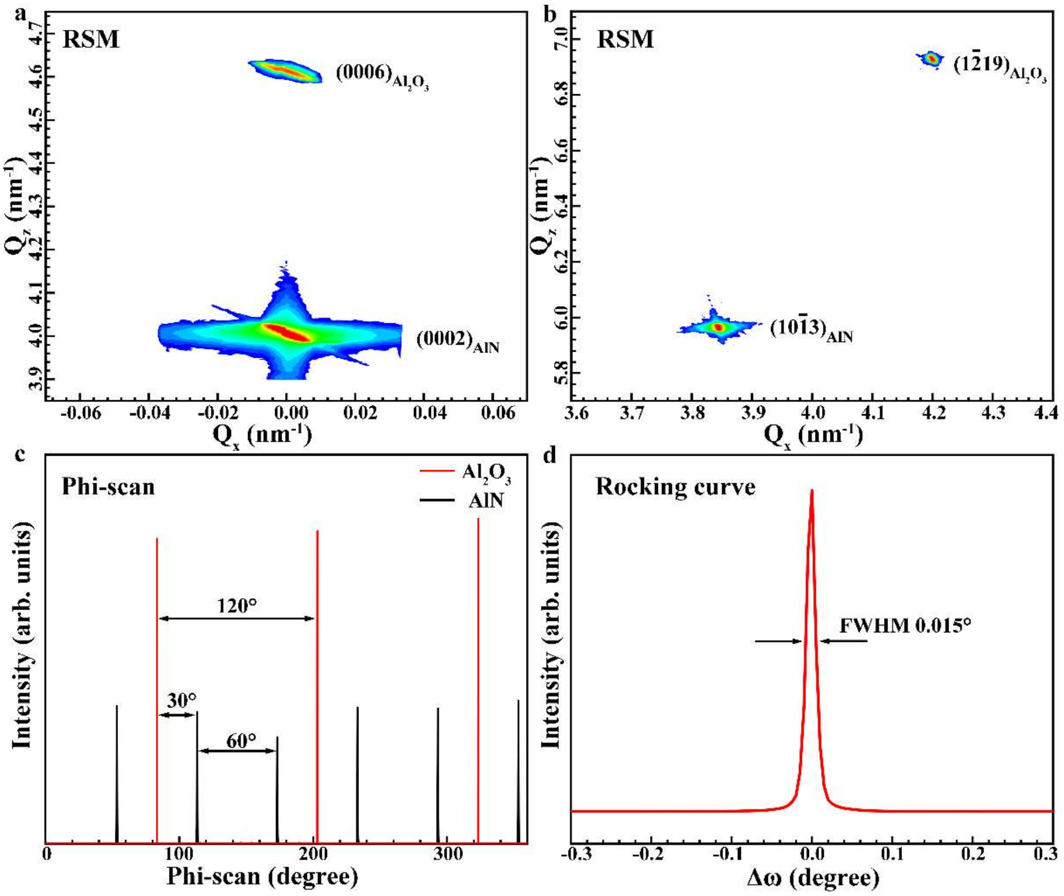

Supplementary Figure S5 | (a,b) HRXRD reciprocal space map (RSM) patterns of the AlN film along the in-plane and out-of-plane directions. According to the RSM patterns, the lattice constants of the AlN film along the out-of-plane direction are consistent with those of perfect AlN crystal, while the in-plane lattice constants are slightly increased. These facts indicate that the AlN film has been fully relaxed along the out-of-plane direction, but has a small in-plane tensile strain. **(c)** Phi-scan patterns of the AlN film and Al_2O_3 substrate. The AlN film grows epitaxially on the Al_2O_3 substrate with 30° in-plane rotation to minimize the lattice mismatch at the interface. **(d)** Rocking curve of the AlN (0002) peak. The FWHM is measured to be 0.015°, suggesting that the AlN film has an extremely high crystallinity.

Supplementary Figure S6

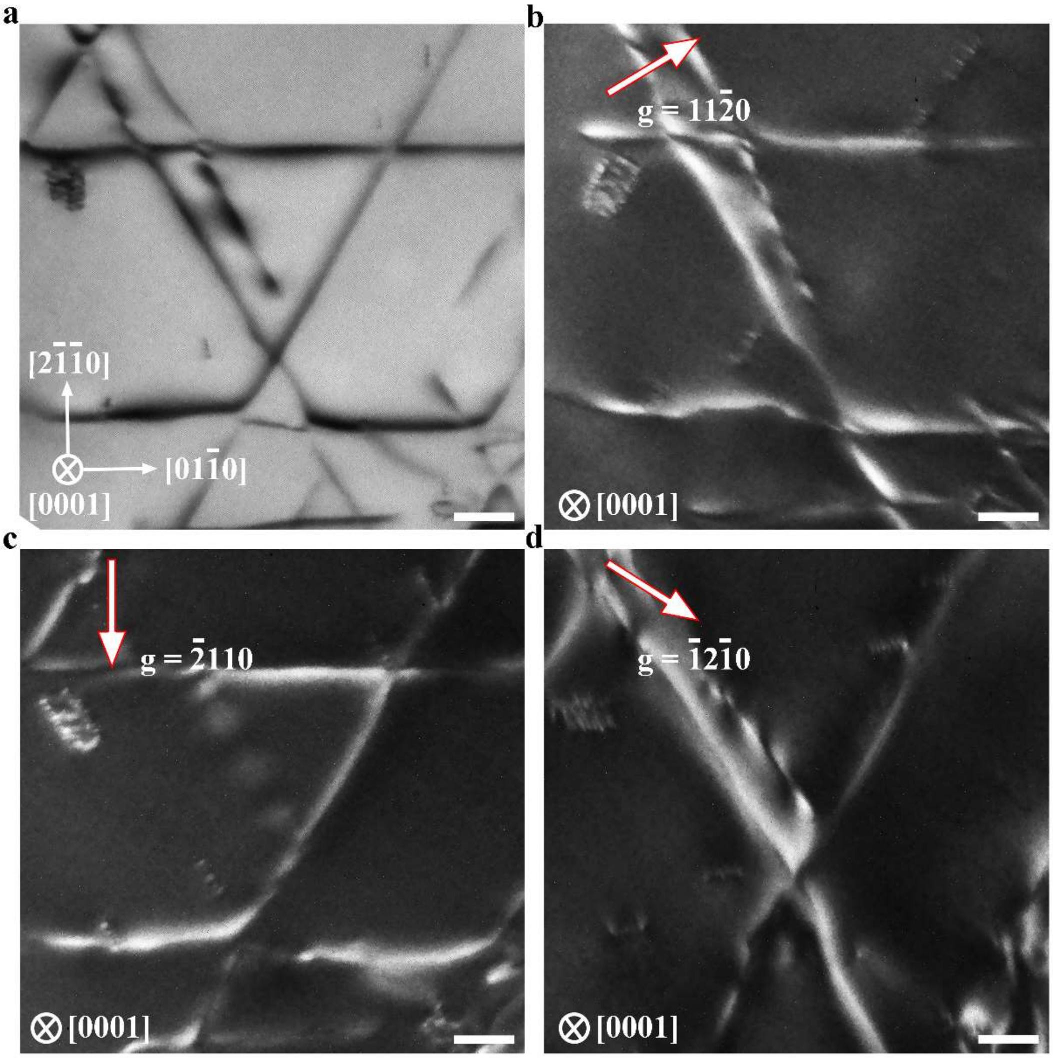

Supplementary Figure S6 | Misfit dislocation networks at the AlN/Al_2O_3 incoherent interface. (a) Bright-field TEM image, **(b-c)** Weak-beam dark-field TEM images recorded at **g** = $11\bar{2}0$ **(b)**, $\bar{2}110$ **(c)**, and $\bar{1}2\bar{1}0$ **(d)**. The Burger vectors of the interfacial misfit dislocations are determined to be 1/3<10$\bar{1}$0>. Scale bar, 50 nm.

Supplementary Figure S7

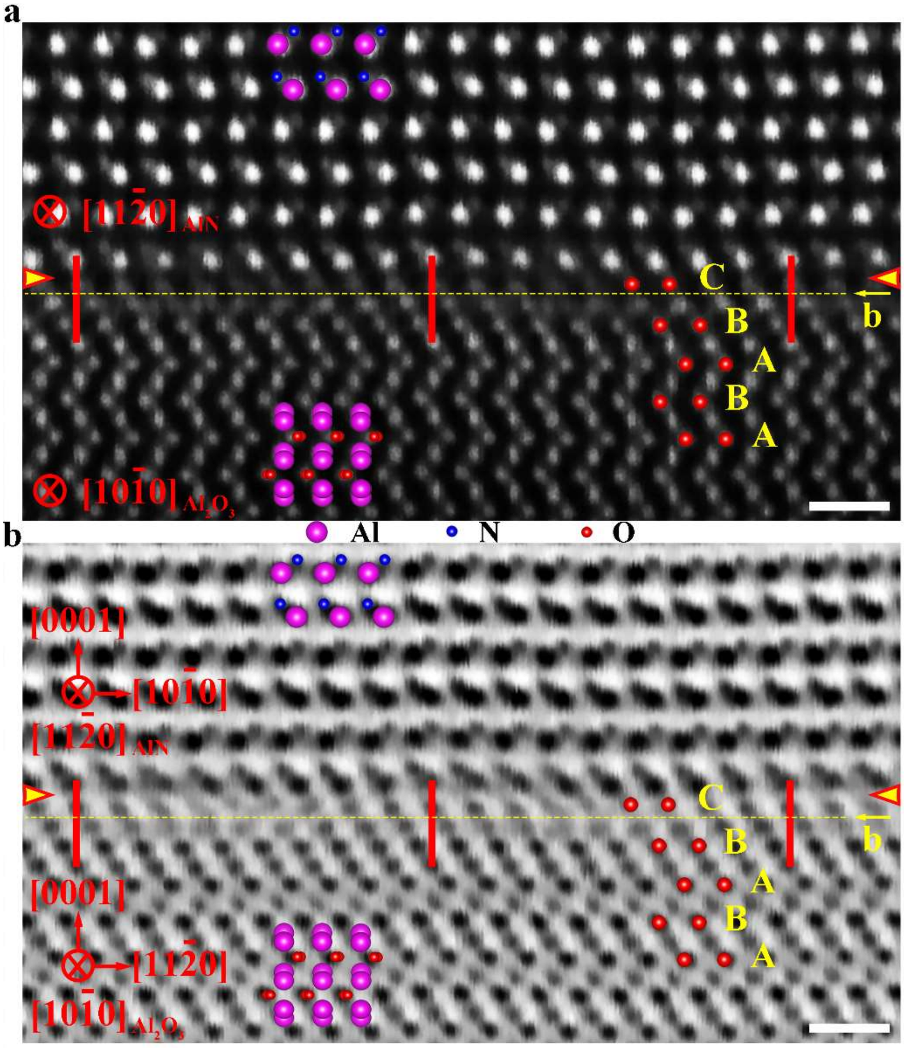

Supplementary Figure S7 | HAADF image showing the stacking fault of region II. (a) HAADF and **(b)** ABF STEM images of type II interface along the [11$\bar{2}$0]AlN//[10$\bar{1}$0]Al_2O_3 zone axis. A, B, and C represent the stacking order of O atomic in Al_2O_3 along the [0001] direction. The stacking fault is indicated by dashed yellow line. Scale bar, 5 Å.

Supplementary Figure S8

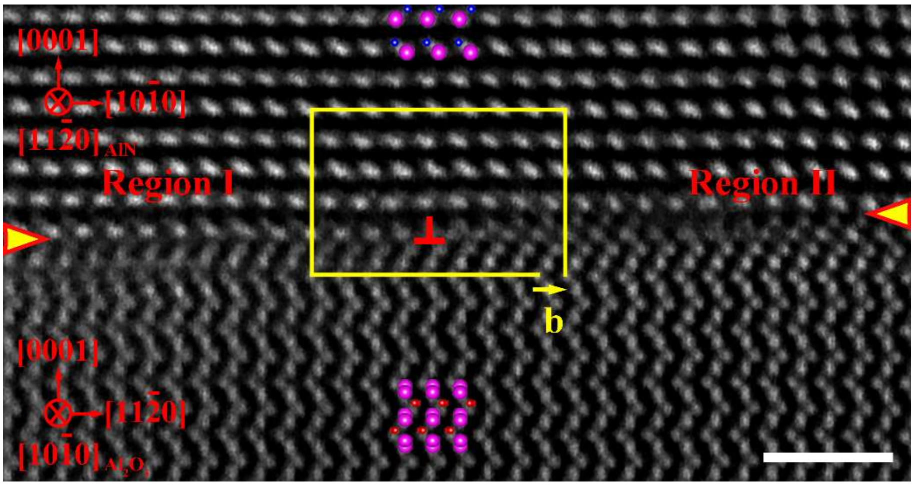

Supplementary Figure S8 | HAADF image showing the transition between the type I and type II interfacial structures. The type I and type II interfacial structures are connected together by a misfit dislocation with a projected Burgers vector of 1/6[11$\bar{2}$0]. The interface is indicated by yellow arrows. Scale bar, 1 nm.

Supplementary Figure S9

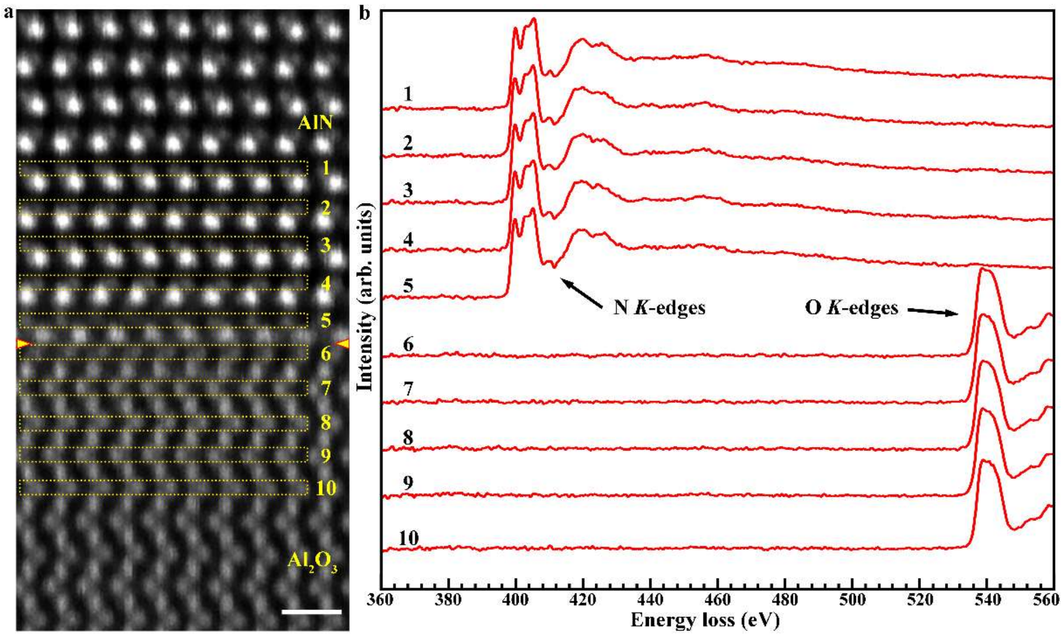

Supplementary Figure S9 | (a) HAADF STEM image of the type I interfacial structure of the AlN/Al_2O_3 incoherent interface. **(b)** Atomic-layer-resolved EELS spectra of the O and N *K*-edges across the interface. The AlN film is terminated with Al plane and the Al_2O_3 substrate is terminated with O plane at the AlN/Al_2O_3 interface. The type I interfacial structure has no detectable anion intermixing. The interface is indicated by yellow arrows. Numbers of 1-10 indicate the regions for EELS measurements. Scale bar, 5 Å.

Supplementary Figure S10

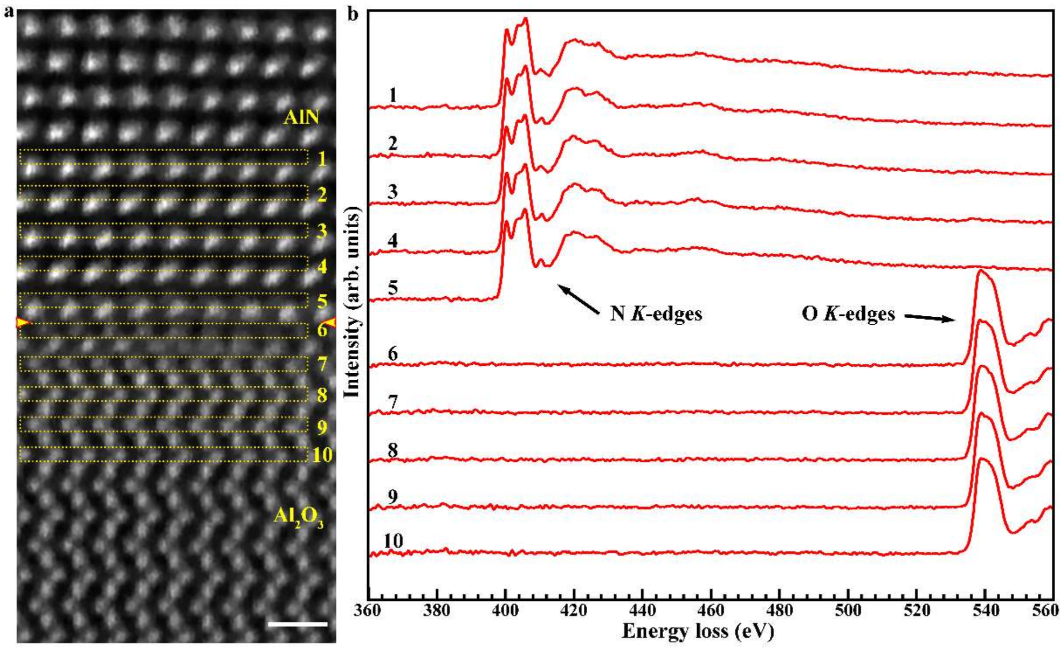

Supplementary Figure S10 | (a) HAADF STEM image of the type II interfacial structure of the AlN/Al_2O_3 incoherent interface. **(b)** Atomic-layer-resolved EELS spectra of the O and N *K*-edges across the interface. The AlN film is terminated with Al plane and the Al_2O_3 substrate is terminated with O plane at the AlN/Al_2O_3 interface. The type II interfacial structure has no detectable anion intermixing. The interface is indicated by yellow arrows. Numbers of 1-10 indicate the regions for EELS measurements. Scale bar, 5 Å.

Supplementary Figure S11

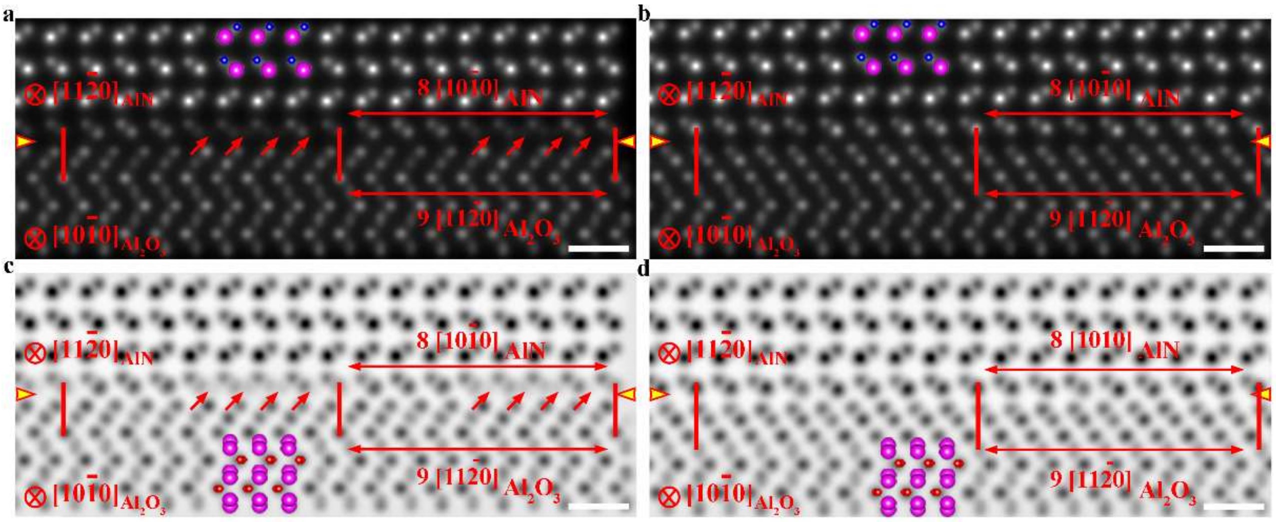

Supplementary Figure S11 | Simulated HAADF and ABF STEM images. Simulated HAADF and ABF STEM images for the type I **(a,c)** and type II **(c,d)** structures at the AlN/Al_2O_3 incoherent interface, respectively. The simulated images are consistent well with the corresponding experimental counterparts. Interfaces are indicated by yellow arrows. Scale bar, 5 Å.

Supplementary Figure S12

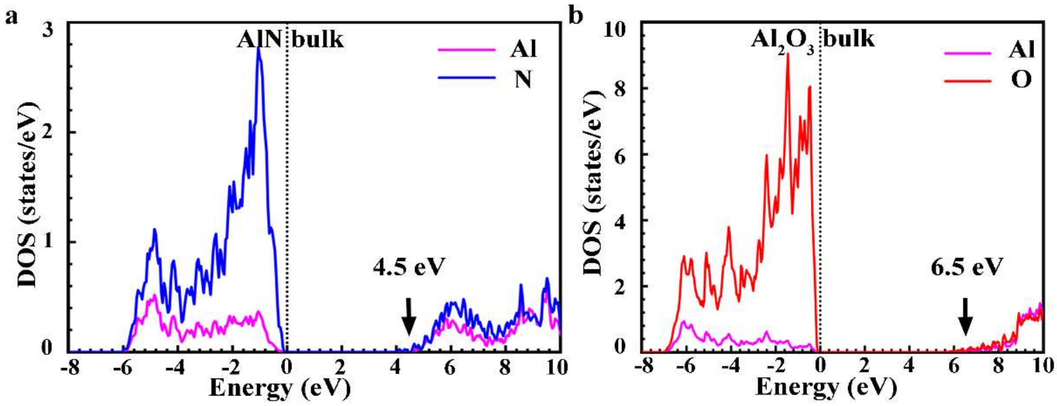

Supplementary Figure S12 | DOS plots of the bulk AlN and Al_2O_3. (a) DOS of the AlN bulk and **(b)** Al_2O_3 bulk. The band gaps of the AlN and Al_2O_3 are 4.5 and 6.5 eV, respectively. The conduction bands minima are marked by black arrows.

Supplementary Figure S13

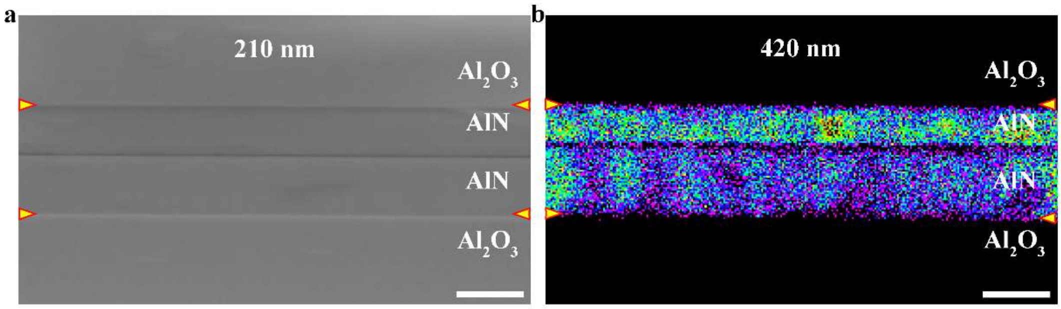

Supplementary Figure S13 | Cathodoluminescence measurements at the AlN/Al2O3 interface. (a) SEM secondary electron image, **(b)** Corresponding CL mapping using the 420 nm laser. The photoexcitation at 420 nm originates from the AlN films. Interfaces are indicated by yellow arrows. Scale bar, 2 μm.
